# An integrated photonic-assisted phased array transmitter for direct fiber to mm-wave links

**DOI:** 10.1038/s41467-023-37103-w

**Published:** 2023-03-14

**Authors:** Pouria Sanjari, Firooz Aflatouni

**Affiliations:** Department of Electrical and Systems Engineering, University of Pennsylvania, Philadelphia, PA 19104 USA

**Keywords:** Electrical and electronic engineering, Integrated optics

## Abstract

Millimeter-wave (mm-wave) phased arrays can realize multi-Gb/s communication links but face challenges such as signal distribution and higher power consumption hindering their widespread deployment. Hybrid photonic mm-wave solutions combined with fiber-optics can address some of these bottlenecks. Here, we report an integrated photonic-assisted phased array transmitter applicable for low-power, compact radio heads in fiber to mm-wave fronthaul links. The transmitter utilizes optical heterodyning within an electronically controlled photonic network for mm-wave generation, beamforming, and steering. A photonic matrix phase adjustment architecture reduces the number of phase-shift elements from *M* × *N* to *M* + *N* lowering area and power requirements. A proof-of-concept 2 × 8 phased array transmitter is implemented that can operate from 24–29 GHz, has a steering range of 40°, and achieves 5 dBm EIRP at an optical power of 55 mW without using active mm-wave electronics. Data streams at 2.5 Gb/s are transmitted over 3.6 km of optical fiber and wirelessly transmitted attaining bit-error rates better than 10^−11^.

## Abstract (web summary)

An integrated phased array transmitter chip that uses an electronically controlled photonic network for millimeter-wave generation and beam formation is developed and used to demonstrate a fiber to wireless communication link.

## Introduction

The electronic beam steering capabilities of phased arrays make it an attractive technology for a wide range of applications ranging from satellite communication^1–3^ to radar systems^4^. To support the continuous growth of mobile traffic and ever-increasing demand for bandwidth, millimeter-wave (mm-wave) phased arrays have also been utilized for 5G wireless and mobile communication networks^5^ given the large bandwidth available around mm-wave carriers and additional spectrum allocated in this regime.

Notwithstanding their utility, the system-level performance of networks employing mm-wave phased arrays can be significantly impacted by atmospheric absorption and link blockage (due to a high degree of penetration loss) necessitating smaller cells and a larger number of access points, while higher power consumption and hardware costs have hindered their proliferation. In order to support densification and provide high-data-rate interconnectivity, fiber to mm-wave fronthaul architectures have previously been proposed^6^. The low propagation loss and high bandwidth available around optical carriers make fiber optic technology a good candidate to address 5G-to-XG fronthaul capacity and density issues. Furthermore, such fiber optic to mm-wave systems can seamlessly interface with existing optical access infrastructure and may exploit the underlying photonics to assist in the processing and generation of mm-wave signals aiding the implementation of cost and energy-efficient high bandwidth transceivers^7,8^.

At the circuit level, mm-wave phased array design challenges include electrical routing, data and clock distributions, crosstalk, and electromagnetic interference which is exacerbated with increasing frequency and array size. On-chip, due to the limited quality factor of the passive components, interconnects suffer from relatively high insertion losses, which are often compensated using active mm-wave blocks leading to higher power consumption. Furthermore, mm-wave passive devices, such as the Wilkinson divider or branch-line coupler, are still typically large and tend to consume most of the chip area^9^. At the board level, LO distribution networks are often complex and lossy and do not scale favorably with size and frequency. Phased array architectures employing on-chip frequency synthesizers^10^ can to some extent circumvent the challenges associated with high-frequency routing on printed circuit boards (PCB) but at the cost of increased per element power consumption and chip area required for LO synthesis and synchronization. Additionally, in such systems, synchronization of the various oscillators in the array necessitates the use of a lower reference frequency which in turn leads to noise multiplication in the phased-locked loop (PLL) of the synthesizer^11^.

Given the availability of low-loss delay lines, the immunity to electromagnetic interference, and the large available instantaneous bandwidth, photonics devices and systems can be leveraged to address some of the limitations of all-electronic phased arrays. Photonic beam-formers for microwave and mm-wave phased arrays have been considered and investigated for many years^12^. In such beamformers, typically, the electrical signal is up-converted to the optical domain (e.g. using an electro-optic modulator) and optically delayed using various true time delay (TTD) techniques (or phase shifted in the case of narrowband applications) to create appropriate delay settings for the different antenna elements. Use of TTD techniques may be particularly advantageous in ultra-wideband phased arrays in order to mitigate beam squint^13^. Notably, designs exploiting chromatic dispersion in fibers^14^, fiber-Bragg grating prisms^15,16^ and chirped fiber gratings^17,18^ used in conjunction with tunable laser sources to constitute wavelength dependent tunable delay lines, have previously been reported. Other designs include discrete TTD beamformers based on switchable fiber delay lines utilizing optical switches^19^ and or demultiplexers^20,21^ and beamformers employing photonic microwave phase shifters^22^ for narrow band applications. However, despite their excellent performance, the widespread adoption of such techniques has often been hindered due to the use of discrete, power-hungry, and bulky components utilized in a benchtop setup.

More recently, with the advancements of CMOS compatible photonic integrated circuits, integrated photonic beamformers have also been devised and reported. In refs. ^23,24^, cascaded optical ring resonators and in ref. ^25^ Mach-Zehnder interferometers with tunable coupling ratios serve as broadband tunable delay lines whereas in ref. ^26^ switchable waveguide delay lines are used to form a photonic beamformer. Such integrated architectures have the potential to enable scalable and high-yield integrated photonic-assisted TTD phased arrays (or timed arrays), which can address some of the challenges of conventional electronic implementations, but often at a cost of increased complexity and chip area due to the use of TTD elements. Moreover, in refs. ^23–26^, active mm-wave devices are used to generate the mm-wave carrier in the electrical domain limiting their operational bandwidth and further increasing the complexity and power consumption as the frequency increases.

In this article, we report the demonstration of an integrated photonic-assisted mm-wave phased array transmitter that uses the principle of optical heterodyning for mm-wave generation and utilizes a low-loss reconfigurable photonic network for beam formation and steering. The integrated photonic-assisted phased array is used to generate and radiate a mm-wave beam carrying a multi-Gb/s data stream at the desired direction without the use of any active mm-wave devices. The data stream is directly transferred from an optical wave propagating through an optical fiber to the mm-wave carrier making the design applicable for low-power, compact radio heads in fiber to mm-wave links. In the proposed architecture, for an *M* × *N* mm-wave phased array, a photonic matrix phase adjustment architecture is used that utilizes *M*+*N* (as opposed to *M* × *N*) phase shifters to perform 2D beam steering significantly reducing chip area and power consumption. The linear superposition of optical waves within a row and column waveguide, at the corresponding intersection point of the matrix architecture, is photodetected generating a mm-wave current due to the non-linear operation of the photodiode on optical electric fields. Optical heterodyning allows the link to utilize the large gain of the lasers to generate highly tunable mm-wave carriers, limited by the bandwidth of the photodiode, while at the same time eliminating costly broadband modulators and electronics required to up-convert the modulated mm-wave for on-chip processing. This approach has the added advantage of being able to phase-shift the electrical signal in the optical domain^27^ rather than the electrical domain where it is more challenging to design high bandwidth and low insertion loss mm-wave phase shifters that support multiple frequency bands^28,29^. Furthermore, since the mm-wave is generated at the remote end, this approach is also beneficial for data transmission over fiber as it is more immune to chromatic dispersion which can result in signal fading^30^. The main concern with optical heterodyning stems from the relatively high phase noise of the generated mm-wave due to the beating of two free-running independent optical sources. To circumvent this issue, given the advances on integrated narrow linewidth lasers^31^, techniques such as optical injection locking^32^ and optical phase-locked loops^33^ can been used for coherent heterodyning to generate low phase noise signals. Alternatively, phase-insensitive detection^34^ techniques can be used in the receiver to relax the linewidth requirements as later demonstrated. The prototype 2 × 8 wideband photonic-assisted mm-wave phased array transmitter chip uses an electronically controlled photonic distribution network consisting of silicon nanowaveguides, directional couplers, grating couplers, photodiodes, and thermal phase shifters for mm-wave generation and beamforming. The array is capable of operating from 24 GHz to 29 GHz limited by the bandwidth of the PCB patch antennas. The transmitter chip achieves an estimated EIRP of 5 dBm at 25.5 GHz (which increases quadratically with optical power) with 55 mW of optical power coupled into the chip and without requiring any active electronic gain blocks. The phased array chip uses 10 thermo-optic phase shifters and achieves a steering range of 40° in elevation (limited only by the on-chip thermo-optic phase shifters) and about 90° in azimuth. A direct fiber to mm-wave fronthaul link is demonstrated, where a data stream is successfully transmitted over 3.6 km of optical fiber and radiated at different angles over a mm-wave wireless link using the photonic-assisted phased array transmitter chip. Data rates as high as 2.5 Gb/s with bit-error rates (BER) better than 10^−11^ are achieved. The 16-element electronic-photonic phased array chip was monolithically integrated in GlobalFoundries GF9WG process within a footprint of 0.9mm^2^. The average power consumption of the chip is around 200 mW, mostly used by the thermal phase shifters, with an additional 30 mW due to the photodiode reverse bias currents when operating at 5 dBm EIRP.

## Results

### Principle of operation

**Fig. 1:**
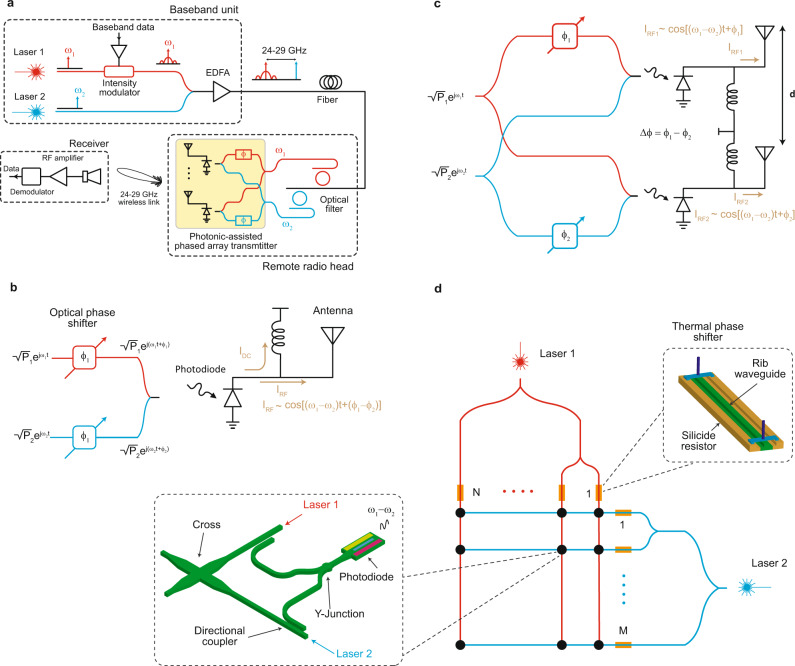
Photonics for mm-wave beam formation and generation. **a** A potential fiber to mm-wave front haul architecture with remote heterodyne detection employing a photonic*-*assisted phased array transmitter. **b** Optical heterodyning scheme used to generate mm-wave signal with the photodiode directly driving an antenna. **c** Illustration of a two-element phased array using optical distribution and beam formation. **d** *M* × *N* matrix architecture with path sharing used to reduce the number of phase-shift elements. The inset on the right shows the architecture of the thermal phase shifter where the central part of a rib waveguide confines the optical mode. The sides of the waveguide are silicided and locally increase the temperature when a current flows through them. The inset on the left shows the photonic network at the junction of a row and column. Directional couplers, with varying coupling lengths, are used to couple light from lasers 1 and 2 ensuring equal power distribution. The two coupled optical waves are then combined using a Y-junction and illuminate a photodiode generating the millimeter wave. The cross allows the waveguides carrying the optical waves from lasers 1 and 2 to overlap with minimal cross talk.

An important application for photonic-mm-wave phased arrays is their deployment in remote radio heads (RRH) of high data-rate networks where data processing and modulation is centralized at a baseband unit (BBU) and delivered to the RRH via optical fiber. Figure 1a is a general illustration of such a network pertaining to the proposed photonic-assisted mm-wave phased array transmitter. Here, a signal laser (laser 1), modulated with multi-Gb/s baseband data, can be combined with a local oscillator laser (laser 2), and remotely sent through an optical fiber to the photonic-assisted phased array transmitter for mm-wave generation, beamforming and steering. The proposed 2 × 8 phased array chip uses the principle of optical heterodyning for mm-wave generation. Consider the case that the electric fields at the outputs of lasers 1 and 2, as shown in Fig. 1b, are written as

1 $$\begin{array}{ccc}{E}_{1}(t)=\sqrt{{P}_{1}}{e} \, ^{j({\omega }_{1}t+{\phi }_{1})} & {{{{{\rm{and}}}}}} & {E}_{2}(t)=\sqrt{{P}_{2}}{e} \, ^{j({\omega }_{2}t+{\phi }_{2})}\end{array}$$

where *P*_*i*_, *ω*_*i*_, and *ϕ*_*i*_ correspond to the optical power, the angular frequency, and the phase of laser *i* (*i* = 1, 2) respectively. When the two lasers are combined and photodetected, the photocurrent is given by

2 $${I}_{{{{{{\rm{PD}}}}}}}=\frac{R}{2}({P}_{1}+{P}_{2})+R\sqrt{{P}_{1}{P}_{2}}\,\cos [({\omega }_{1}-{\omega }_{2})t+({\phi }_{1}-{\phi }_{2})]$$

where *R* is the responsivity of the photodiode. As evident from Eq. (2), the optical phase is preserved, and the phase of the generated RF signal can be controlled by adjusting *ϕ*_1_ and *ϕ*_2_. This is similar to the LO phase-shifting technique that is often used in all-electrical phased arrays^35^. The photodiode can then be used to directly drive an antenna^36,37^. In this case, the RF photocurrent flows into the antenna where an electromagnetic wave will be radiated with an emitted power that also depends on the radiation resistance and efficiency of the antenna.

Figure 1c shows the block diagram of a two-element antenna array where light from the lasers is equally distributed between two photodiodes directly driving antennas with a pitch of *d*. By controlling the relative phase between the antenna elements *Δϕ* = *ϕ*_1_−*ϕ*_2_, which is done using optical phase shifters, the radiated beam can be steered to the direction sin(*θ*_beam_) = *Δϕ*/2*πd*, where *λ* corresponds to the wavelength of the generated mm-wave^38^. The architecture in Fig. 1c can be extended to a 2D phased array, where a photonic distribution network is used for mm-wave generation, beamforming, and steering.

### Architecture of the photonic-assisted mm-wave transmitter

A unique advantage of a photonic distribution network is depicted in Fig. 1d where a path-sharing matrix architecture is utilized for the reported *M* × *N* photonic-assisted mm-wave phased array. While *M* × *N* arrays with individual phase and amplitude control can enable flexible beam shaping, multi-beam formation and apodization^39^, for single beam formation and steering, the matrix architecture shown in Fig. 1d requires *M* + *N* phase shifters (as opposed to *M* × *N*), which significantly reduces both chip area and power consumption. At each row-column intercept point, the linear superposition of electric fields of the optical waves is photodetected (corresponding to a non-linear operation on electric fields) to generate the mm-wave photocurrent. By controlling the optical phase in the columns (rows), the relative phase between adjacent elements in the *X* (*Y*) direction can be controlled thus enabling independent steering of the generated beam in both directions. The path-sharing matrix architecture used in the phased array transmitter looks similar to a one previously used for an optical phased array^40^ but the beamformation principles, beam steering capability and the design methodology pertaining to signal routing, power and phase distribution is fundamentally different. Further design details are provided in Supplementary Note 1.

**Fig. 2:**
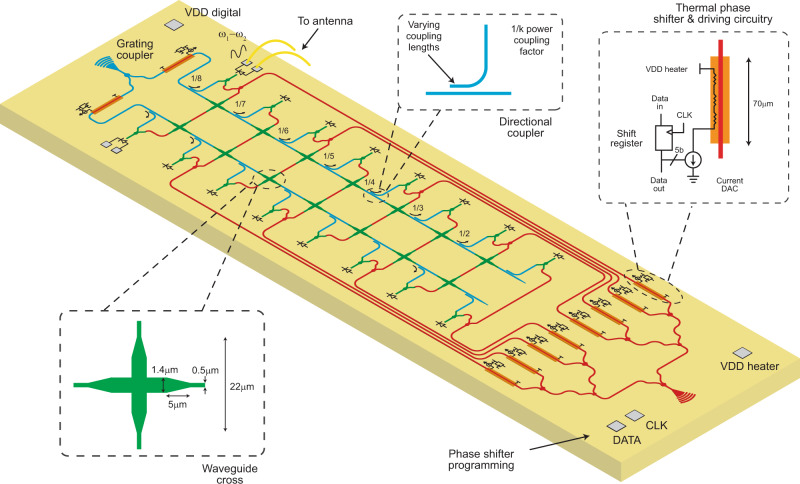
Integrated electronic-photonic phased array transmitter chip. Architecture of the 2 × 8 photonic*-*assisted mm-wave phased array transmitter chip fabricated in GlobalFoundries 90 nm CMOS 9WG process. The transistor level schematics of the electronic circuitry used to drive and program the thermal phase shifters are shown in Supplementary Note 6.

In the implemented 2 × 8 architecture (Fig. 2), 16 SiGe photodiodes are arranged on two rows and eight columns. Lasers 1 and 2 are coupled into the electronic-photonic phased array chip using grating couplers. Light coupled into the chip from laser 1 is divided equally into eight branches using three layers of Y-junctions with thermo-optic phase shifters placed in each branch. Each branch is then distributed to two adjacent photodiodes on a column using a Y-junction. Light from laser 2 is also equally divided into two arms using a Y-junction with a thermo-optic phase shifter placed on each arm. Each arm is then distributed to eight adjacent photodiodes on a row using a series of directional couplers with varying coupling lengths to ensure equal power distribution. In the absence of multi-layer optical interconnection, compact optical waveguide crossings with insertion losses of around 0.1 dB and crosstalk of around −30 dB allow flexible routing for the two different laser paths. At the intersection of each row and column, optical waves from the two lasers are combined using a Y-junction and photodetected to generate the mm-wave signal. Characterization details of the photonic network related to optical power distribution among the photodiodes can be found in Supplementary Note 2. Each of the 10 thermo-optic phase shifters, implemented as a silicide resistor (resistor heaters) and placed in close proximity to the waveguide, is independently driven by an on-chip 5-bit digital to analog converter (DAC). Each DAC converts the digital content of a corresponding shift register, serving as the per phase shifter memory cell, and can be serially programmed through off-chip data and clock signals.

**Fig. 3:**
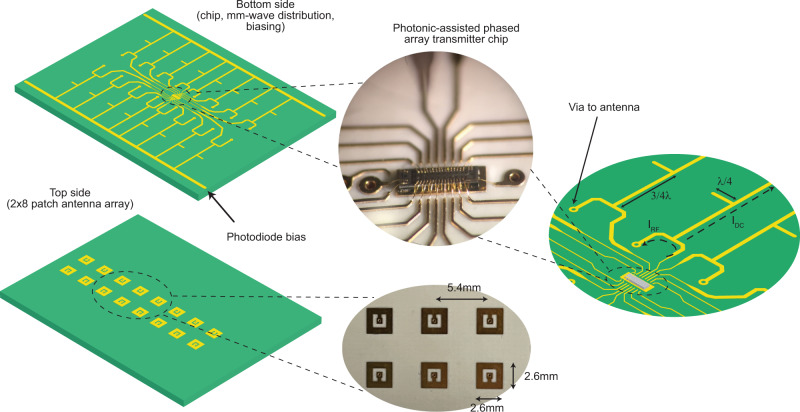
PCB with embedded antennas. 4-layer PCB with 2 × 8 array of patch antennas on the top side and the mm-wave distribution network and the photonic*-*assisted mm-wave phased array chip on the bottom side. Power, CLK (Clock) and DATA lines, used to program thermo-optic phase shifters, are routed on the inner layers. The electronic-photonic chip was backgrinded from 750μm to 250μm to reduce the effect of the wirebonds.

The photocurrents (at the output of on-chip photodiodes) are wire-bonded to a PCB (Fig. 3) and routed to 16 center-fed patch antennas^41^ that are placed 0.54 mm apart (∼0.5*λ* at 28 GHz). The 2 × 8 array of patch antennas is placed on the top side of the PCB whereas the chip and mm-wave distribution network feeding the antennas and biasing the photodiodes are placed on the bottom side. The on-PCB mm-wave distribution network is based on microstrip transmission lines. Biasing for the photodiodes is provided through the distribution network on *λ*/4 lines that convert a low impedance (at the biasing point) to a high impedance (to avoid loading the transmission line). The design and simulation of the PCB, including the antennas and mm-wave distribution network, are further discussed in Supplementary Note 3.

### System measurements

**Fig. 4:**
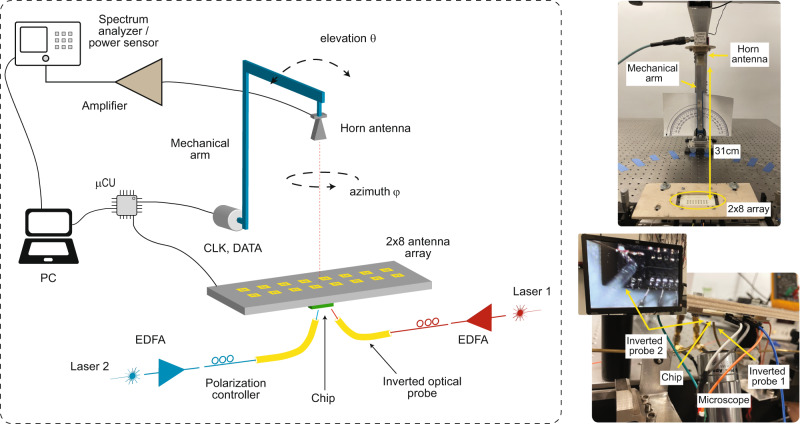
Beam formation and radiation pattern measurement setup. Measurement setup used to calibrate the phased array and measure the radiation pattern when steered to different angles. The steering angle is set by programming the on-chip thermo-optic phase modulators through a CLOCK and DATA bit stream which are generated externally by a microcontroller.

As previously discussed, the relative phase between the antenna elements, which are driven by the photodiodes, is determined by adjusting the optical phases in the nanophotonic waveguides. Note that even with symmetric waveguide structures and optical path matching, the variation of the effective refractive index across the chip attributed to process variation^42^ can result in phase mismatch between the array elements. However, these are static phase errors and a calibration process can provide the necessary corrections. Figure 4 illustrates the measurement setup used to calibrate the phased array and measure the radiation pattern. A mechanical arm with an attached horn antenna was used to scan the surface of a half sphere (in elevation and azimuth angles). A spectrum analyzer was used to measure the received signal from the horn antenna.

**Fig. 5:**
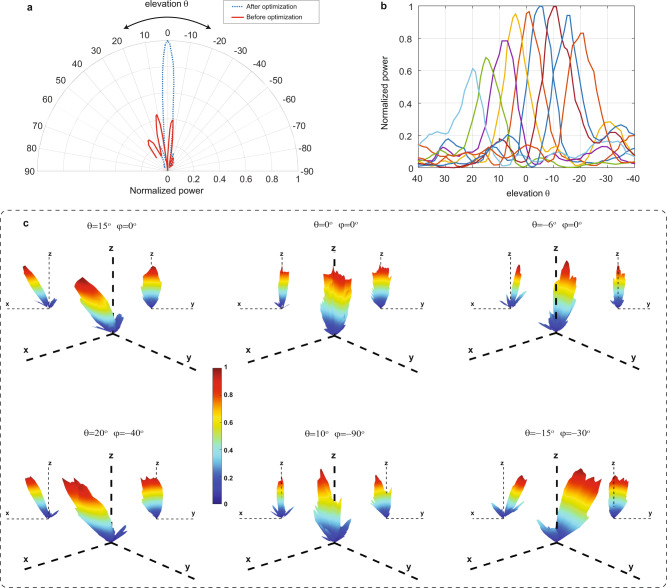
Beam steering and radiation pattern measurement. **a** 1D radiation pattern (*φ* = 0° plane) before and after calibration with the receiver horn antenna placed broadside to the array. **b** Demonstration of 1D beam steering (*φ* = 0° plane, *f* = 25.5 GHz). **c** 3D radiation pattern with the phased array configured to point at various angles.

Figure 5a shows the 1D radiation pattern before and after the calibration process with the receive horn antenna placed broadside to the array (*θ* = 0°, *φ* = 0°) during the optimization. A similar calibration process is carried out at different angles (both *θ* and *φ*) and the results are saved to form a look-up table (LUT) that can be used to steer the phased array to the desired angle. It is worth pointing out that while ambient temperatures changes can induce excess phase in the nanophotonic waveguides, its effect on the radiation pattern and LUT can be mitigated, to a certain extent, through path length matching. In this scenario, the induced excess phase is common to the waveguides and as such the relative phase difference, which sets the radiation pattern, remains largely unaffected. Having said that, large temperature shifts may require the LUT to be adjusted if the temperature shift drastically changes the thermo-optic coefficient or the thermal conductivity between the waveguides on the buried oxide and the silicon substrate. The results of ref. ^43,44^ seem to suggest that the thermo-optic coefficient of silicon remains relatively constant between 200-300 K while the thermal conductance of thin silicon films on SOI varies by around 30% in the range of 250-350 K.

Figure 5b demonstrates beam-steering measurement results where for *φ* = 0°, a scanning range of ±20° is achieved in elevation *θ* (as cross-polarization levels are <−15 dB they are omitted for clarity). Figure 5c shows the measured 3D radiation pattern of the phased array for different elevation and azimuth angle settings. Note that in Fig. 5b, the limited scanning range and the asymmetry in the beam powers (<1.3 dB between the positive and negative elevation angles) primarily stem from the low efficiency of the thermo-optic phase shifters in the GlobalFoundries GF9WG process which make the task of setting ideal phases for all the elements more difficult due to the large electrical power required to induce a *π* phase-shift (*P*_π_ ∼ 60 mW—Supplementary Note 4). This issue could be improved in future designs by using more efficient thermo-optic phase shifters with undercuts^45^ or by using capacitive phase modulators with near-zero static power consumption^46,47^. The latter approach can also eliminate thermal crosstalk between phase shifters which may adversely affect the phase adjustment capability.

**Fig. 6:**
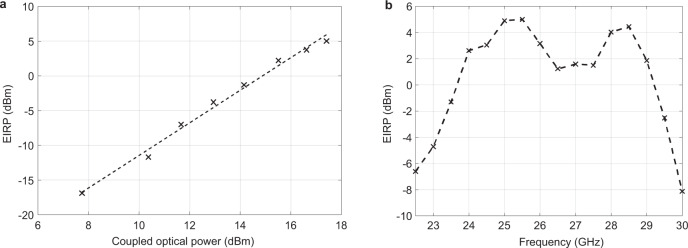
EIRP measurement. **a** EIRP increasing quadratically with input optical power. **b** EIRP vs heterodyne frequency.

The EIRP was calculated by measuring the power received. Figure 6a shows that as an advantage of the proposed architecture, the EIRP increases quadratically with optical power achieving a value of around 5 dBm with an optical power of around 55 mW coupled into the chip. It should be noted that the quadratic increase will eventually be limited by the photodiode compression (Supplementary Note 5) and two-photon absorption (TPA)^48^ in the silicon waveguides.

Figure 6b shows the measured EIRP vs the operating frequency demonstrating the wideband operability of the phased array. Note that the bandwidth of the system is limited by the bandwidth and radiation efficiency of the patch antennas, while the upper limit of the electronic-photonic chip is set by the bandwidth of the photodiodes which in this process is around 42 GHz (Supplementary Note 5)^45^.

**Fig. 7:**
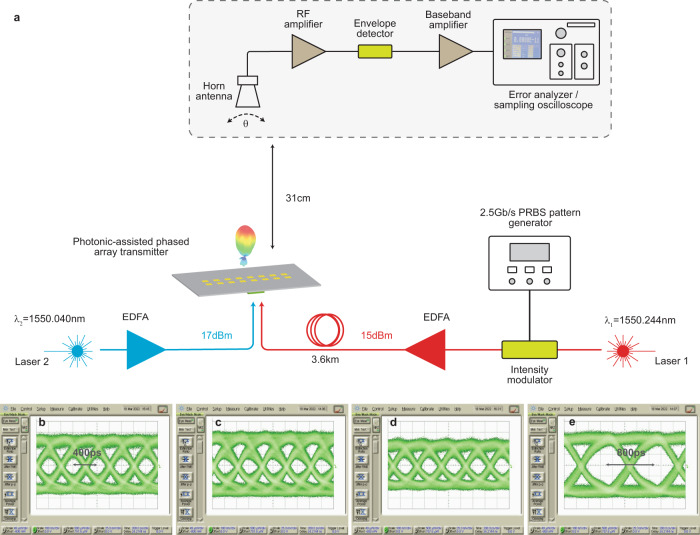
Optical and wireless data transmission. **a** Measurement setup of an optical fiber to mm-wave wireless link. The eye diagrams of the detected data streams (at 2.5 Gb/s) after traveling over 3.6 km of optical fiber and wireless transmission at the mm-wave with the phased array steered to **b** *θ* = 12°, **c** *θ* = 0° and **d** *θ* = −15°. **e** Eye diagram of the detected 1.5 Gb/s signal with BERs < 10^-11^.

The implemented photonic-assisted mm-wave phased array chip may be employed for fiber to mm-wave links. In such systems, it is often desired to process the baseband signal at a centralized location and use optical fibers to distribute the high data-rate (e.g. at multi-Gb/s or higher) information to low-cost remote antenna units for wireless transmission. Figure 7a shows the measurement setup of an optical and wireless link used to test the photonic-assisted phased array transmitter chip. One of the optical signals is modulated in on-off key (OOK) format with a non-return to zero (NRZ) pseudo-random bit stream (PRBS-7) pattern using an optical intensity modulator. The modulated optical wave is then amplified using an erbium-doped fiber amplifier (EDFA) and passed through a 3.6 km fiber before interfacing with the chip. The other optical source is used as a local oscillator and down-converts the signal laser inside the chip to produce a modulated mm-wave signal at the desired carrier frequency. The information in the LUT is loaded onto the DACs to form a beam. On the receiver end, a diode-based envelope detector is used to directly demodulate the wirelessly received mm-wave signal and an error analyzer is used to calculate the bit-error rate (BER). Figure 7b–e show the eye diagrams of the demodulated received signal for various configurations. With the carrier frequency set to 25.5 GHz and different steering angles (*θ* = −15°, 0°, 12°), BER < 10^−11^ is measured for data rates as high as 2.5 Gb/s. With the constructed antenna measurement setup (shown in Fig. 4), where the receiving horn antenna is attached to a mechanical arm scanning the surface of a sphere, the wireless transmission distance was limited to 31 cm. However, given the achieved BER and an EIRP of 5 dBm, the maximum wireless transmission distance is estimated to be around 7 m (further details can be found in the Methods section).

## Discussion

Photonic-assisted phased arrays have the potential to address many of the challenges associated with conventional all-electronic phased arrays as the size and frequency of operation increase. In the context of a scalable 5G-XG fronthaul, photonic integrated circuits can be crucial in the widespread adoption of cost and energy-efficient transceivers especially given that the shrinking cell sizes of the network will require an increasing number of radio heads. However, for practical application and successful deployment, a few avenues must be explored.

The power consumption of the implemented chip is predominantly determined by the thermal phase shifters. Although optimization of these devices^49^ can result in lower *P*_π_, large power savings can be expected by replacing the resistive heaters with silicon-insulator-silicon (SISCAP)^46^, metal-oxide semiconductor capacitor (MOSCAP)^47^, PN^50^ or PIN^51^ phase shifters. Compared to heaters, such phase shifters not only have lower static power consumption but also much faster response times (bandwidth) that could enable applications such as rapid beam scanning. Such elements may have a larger footprint and will typically require higher voltages to induce the equivalent phase shift; however, floating body CMOS transistors with transistor stacking techniques can be utilized and monolithically implemented to drive these elements. In this case, the power consumption of the photonic-assisted transmitter will be set by the reverse bias voltage and photocurrents of the photodiodes. To further decrease the power consumption, the reverse bias voltage of the photodiodes may be lowered. Here, the concern may be a reduction in the bandwidth of the photodiode and the RF power compression point. However, bandwidth and RF power compression measurements of the photodiode, shown in Supplementary Note 5, indicate that lowering the reverse bias voltage from 1.5 V to 0.5 V only reduces the bandwidth from around 44 GHz to 40 GHz. Also, the photocurrent at which the RF power compresses by 1 dB reduces from 1.75 mA to 1.4 mA. As a reference point, with 55 mW of optical power coupled to the chip corresponding to an EIRP of 5 dBm, each photodiode draws 1.2 mA of photocurrent from a 1.5 V supply. Thus, further power savings can be expected by lowering the reverse bias voltage without sacrificing much system performance. Note that for fiber to mm-wave links, the lasers and EDFAs which provide a large portion of the power can be located kilometers away at a centralized office or base station and have less stringent requirements with respect to power, area, etc.

To increase EIRP for long-range transmissions, more efficient grating couplers, lower loss photonics devices, and higher optical powers should be considered. High optical powers can be facilitated to some extent by using multiple photodiodes in parallel to increase the compression point. If needed, inductive gain peaking^52^ and tuning techniques may also be employed to increase the bandwidth to compensate for the effects of increased capacitance due to using multiple devices in parallel. Multiple optical inputs and or rib waveguides with higher power handling^53^ can also reduce excess loss in the waveguides at high optical powers (due to non-linear processes such as TPA and TPA induced free carrier absorption). Uni-traveling-carrier (UTC) photodiodes, which have higher power handling and output powers, may also be utilized to achieve higher radiated powers and can be heterogeneously integrated with silicon photonics^54,55^. Alternatively, to achieve an even higher output power per channel, while the complex task of routing, mm-wave generation and beamforming is performed in the photonic domain, trans-impedance and power amplifiers (monolithic or heterogeneous integration) could be utilized after the photodiodes to drive the antennas. Note that the losses of photonic waveguides (1-1.5 dB/cm), Y-junctions (0.5 dB) and optical phase shifters (0.25 dB/mm for silicide resistor heater, 6.5 dB/mm for SISCAP with *V*_π_*L* < 2 V.mm^46^) are typically lower when compared to mm-wave transmission lines (0.5–1 dB/mm on-chip), power dividers (>1dB^56^) and phase shifters (6.5 dB for 5 bit passive switched-LC^57^). Moreover, the loss of these electronic components increases rapidly with increasing frequency whereas the losses of the photonic distribution scheme remain virtually unchanged. As such, while the overall bandwidth of the system is limited by the bandwidth of the mm-wave patch antennas, the photonic chip can be used across a wide range of frequencies as long as the frequency of operation remains within the bandwidth of the photodiodes.

In order to scale to larger arrays, special considerations must be given to the packaging. In cases where wire bonding does not satisfy the pitch requirements, more sophisticated 3D integration solutions such as through-silicon vias and/or flip-chip bonding along with on-PCB antennas^58^ or antenna-in-package^59^ designs may be required, while to couple light to a flip-chip bonded photonic chip, efficient through-substrate grating couplers^60,61^ or edge couplers will have to be used. Such PICs will then be compatible with tiling-based architectures exploiting modular designs^62^ that are commonly used for large-scale arrays.

The proliferation of photonic-assisted transmitters will be aided by advancements in low-cost, compact integrated semiconductor lasers. In recent years, hybrid, and heterogeneous integration of III-V gain material with silicon and silicon nitride integrated external cavities have yielded lasers with sub-KHz linewidths and moderately high output powers^63–66^. Through optical heterodyning of such lasers, mm-wave signals with high spectral purity and low phase noise can be generated which in turn allow for complex modulation schemes and ultimately higher data rates.

In this work, we have reported an *M* × *N* (2 × 8) photonic-assisted mm-wave transmitter chip based on the principle of optical heterodyning and photonic-assisted beamforming and steering. The electronic-photonic chip consists of a matrix optical distribution network used for optical beamformation which reduces the required number of phase-shift elements from *M* × *N* to *M* + *N* minimizing both area and power consumption. The phased array transmitter achieves an EIRP of 5 dBm at 25.5 GHz with 55 mW of coupled optical power without requiring any active electronic gain blocks and has a 40° steering range. An optical fiber to mm-wave fronthaul link is demonstrated and used to successfully transmit a 2.5Gbaud data stream at various angles using the photonic-assisted mm-wave transmitter.

## Methods

### Beam formation

Figure 4 is an illustration of the measurement setup used to calibrate the phased array and measure the radiation pattern. Two C-band lasers, with a combined linewidth of around 100 kHz, were tuned such that the frequency of their beat-note was set between 24 GHz and 29 GHz (corresponding to the desired frequency of the mm-wave output) and coupled into the electronic-photonic integrated chip through grating couplers using optical probes after polarization adjustment. Light from each grating coupler is distributed equally to 16 photodiodes with thermal phase shifters placed in select paths as described in the matrix architecture of Fig. 1d and shown in Fig. 2. The loss of the network is around 9.5 dB with 5 dB attributed to the grating coupler, 3 dB due to the combiner before the photodiode and 1.5 dB from the excess loss of the splitters and phase shifters. A horn antenna with a gain of 10 dBi, attached to a mechanical arm, was placed 31 cm away in the far field of the antenna array to receive the signal. The output of the horn antenna was then amplified by 35 dB and connected to a spectrum analyzer. In order to improve the radiation pattern and form a beam, a calibration was performed to find the DAC settings that maximize the received power. In this case, the received power on the spectrum analyzer was monitored while the integrated DACs that control the thermo-optic phase shifters were sequentially swept. For each DAC, the code that maximizes the peak power was saved and serially loaded to the on-chip register. Measurements show that typically two to three iterations over the 10 DACs were sufficient for successful beam formation. The pseudocode of the simple calibration algorithm is provided in Supplementary Note 7. To measure the radiation pattern, the mechanical arm with the attached horn antenna was used to scan the surface of a sphere in elevation and azimuth. The received power is measured at each point using the spectrum analyzer. More details on the pattern measurement are provided in Supplementary Note 8.

### EIRP calculation

The EIRP (expressed in dBm) was calculated by measuring the received power *P*_R_ and using the equation

3 $${{{{{\rm{EIRP}}}}}}={P}_{{{{{{\rm{R}}}}}}}-{G}_{{{{{{\rm{R}}}}}}}+{L}_{{{{{{\rm{S}}}}}}}$$

where *G*_R_ and *L*_S_ are the gain of the receiver and free space propagation loss respectively. Measurements were carried out using a diode waveguide power sensor and a 10 dBi horn antenna with cable losses also accounted for to accurately calculate the gain of the receiver. The horn antenna was placed 31 cm away from the transmitting PCB antenna array corresponding to a path loss of around 50 dB.

### Optical and wireless transmission link

Figure 7a is the measurement setup of an optical and wireless link used to test the photonic-assisted phased array transmitter chip. A pattern generator modulated one of the laser signals with NRZ PRBS7 data using a 10 Gb/s optical intensity modulator. The modulated optical signal was then amplified to around 15 dBm using an EDFA and connected to a 3.6 km long single-mode optical fiber. The other optical signal was amplified to around 17 dBm using another EDFA. Inverted optical probes were used to couple the light into the chip via the grating couplers after polarization adjustment. The received mm-wave signal was directly demodulated using a diode-based envelope detector, amplified, and monitored on an error analyzer (sampling oscilloscope) to measure (capture) the bit-error rate (eye-diagram).

### Wireless transmission distance

The minimum detectable signal *P*_MDS_ (in dBm) at the receiver input can be estimated using^67^

4 $${P}_{{{{{{\rm{MDS}}}}}}}=10{\log }_{10}\left(\frac{kT}{1{{{{{\rm{mW}}}}}}}\right)+{{{{{\rm{NF}}}}}}+10{\log }_{10}({{{{{\rm{BW}}}}}})+{{{{{{\rm{SNR}}}}}}}_{\min }-{G}_{{{{{{\rm{A}}}}}}}$$

where *k*, *T*, NF, BW, SNR_min_ and *G*_A_ are the Boltzmann constant, temperature, noise figure of the receiver, bandwidth of the signal, minimum signal to noise ratio required by the receiver and antenna gain respectively. Given a temperature of 300 K, a bandwidth of 2 GHz, a noise figure of 5 dB, an antenna gain of 10 dBi and assuming a SNR_min_ of 13 dB, the minimum required signal level is around −73 dBm. In this case, with an EIRP of 5 dBm, the maximum transmission distance at 25.5 GHz is estimated to be around 7 m corresponding to a path loss of 78 dB.

A list of measurement instruments and equipment is included in Supplementary Table 1.

## Supplementary information

Supplementary Information

## Data Availability

The data that support the findings of this study are available from the corresponding author upon request.

## Code availability

The code used in the present work is available from the corresponding author upon request. The pseudocode of the beam formation optimization process is shown in Supplementary Note 7.
