# Supplementary material for: An integrated photonic-assisted phased array transmitter for direct fiber to mm-wave links
Source: Nat Commun. 2023 Mar 14;14:1414. doi: 10.1038/s41467-023-37103-w (PMC10014951; doi:10.1038/s41467-023-37103-w)
Supplement: Supplementary file 1 — Supplementary Information [file 41467_2023_37103_MOESM1_ESM.pdf]

Supplementary Information

An Integrated Photonic-Assisted Phased Array Transmitter for Direct Fiber to mm-Wave Links

Pouria Sanjari[1] **and Firooz Aflatouni**[1,*]

[1]Department of Electrical and Systems Engineering, University of Pennsylvania, Philadelphia, PA 19104, USA
[*]firooz@seas.upenn.edu

Supplementary Note 1: Path-sharing matrix architecture of the photonic distribution network

Supplementary Fig. 1 shows the general matrix architecture of the optical distribution network used in implementing the photonic-assisted phased array transmitter. The matrix architecture looks similar to the one used in an optical phased array[1]; however, as the beam formation principles and the design methodology regarding routing and power and phase distribution is fundamentally different, the details are presented here.

The light emanating from a laser operating at angular frequency of ω_1 (ω_2) is distributed equally to M (N) rows (columns). The electric field at the input of the mth row (n^{th} column) can be expressed as $e^{j(\omega_1 t-\phi_m)}$ ($e^{j(\omega_2 t-\theta_n)}$) where ϕ_m (θ_n) is the optical phase at the input of the mth row (n^{th} column). Note that for simplicity, the amplitude of the input electric field is considered to be unity. Before the junction of a row and column, directional couplers with varying lengths tap off light from the bus waveguides which are linearly combined using a Y-junction and routed to a photodiode (PD). The coupling lengths of the directional couplers are varied so as to ensure equal amounts of power are tapped off before each junction. As such, the electric field incident on the (m,n) photodiode can be written as

$$E_{m,n} = \frac{1}{\sqrt{2M}} e^{j(\omega_1 t-\phi_m)} + \frac{1}{\sqrt{2N}} e^{j(\omega_2 t-\theta_n)}. \tag{1}$$

Accordingly, the photocurrent (which is a nonlinear function of the electric field of the light to be photodetected) at the output of the photodiode will have a DC and an mm-wave component and is given by

$$I_{m,n} = R(E_{m,n}E^*_{m,n}) = I_{DC} + i_{m,n}, \tag{2}$$

where R is the responsivity of the photodiode and

$$i_{m,n} = \frac{1}{\sqrt{MN}} \cos[(\omega_2-\omega_1)t + \theta_n - \phi_m], \tag{3}$$

indicating the generation of an electrical signal with frequency $\omega_2-\omega_1$ and phase $\theta_n-\phi_m$. Hence, the relative phase of the photocurrents between two adjacent photodiodes on row m will be $\Delta\Theta^m_{n,n+1} = \theta_n - \theta_{n+1}$ and the relative phase between the photocurrents of two adjacent photodiodes on column n will be $\Delta\Phi^n_{m,m+1} = \phi_m - \phi_{m+1}$. By setting $\Delta\Theta^m_{n,n+1} = \Delta\theta$ and $\Delta\Phi^n_{m,m+1} = \Delta\phi$, or in other words setting the relative phase shift between elements on a row (column) to be $\Delta\theta$ ($\Delta\phi$), a beam that can be steered in both

directions will be formed using $M+N$ as opposed to $M\times N$ phase shifters. Note that in the presence of relative phase mismatch between adjacent elements, arising for example due to process variation across the chip, the sidelobe suppression decreases, and power in the main lobe drops. In the implemented scheme employing $M+N$ phase shifters, this effect can be largely compensated through the optimization and calibration process described in the main text and as shown by the measurement results of Fig. 5a. Note that in phased arrays with per element amplitude and phase control, in theory, the effect of the mismatch can be fully compensated. The photonic structures such as the couplers, Y-junctions, and waveguide crosses used in the photonic distribution network were designed and simulated using Lumerical FDTD.

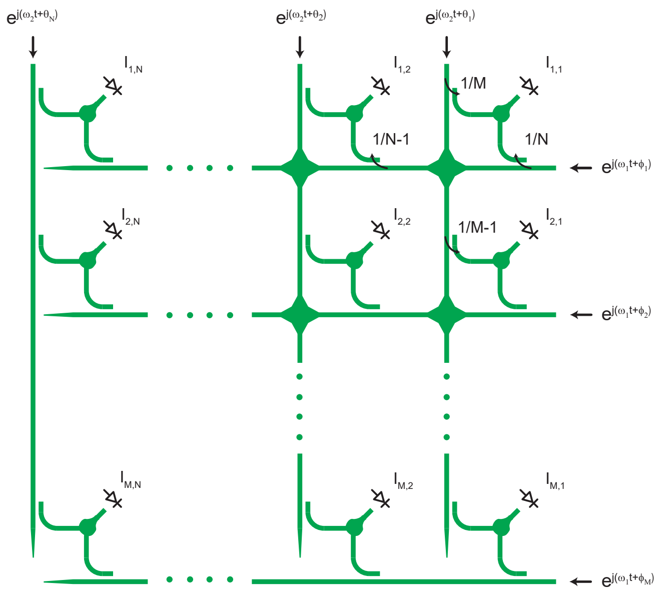

Supplementary Fig. 1 | Path sharing matrix architecture. An optical distribution network utilizing path sharing and a reduced number of phase control elements for mm-wave beam formation and steering. At each intersection point, the linear superposition of the two optical waves is photodetected resulting in an mm-wave current whose phase can be set optically.

Supplementary Note 2: Optical power distribution

A photonic network is used to distribute the laser powers equally into the array of photodiodes. Light from one laser path (red - column) utilizes 4 layers of Y-junction splitters and a Y-junction combiner while the other laser path (blue - row) uses one layer of Y-junction, cascaded directional couplers with varying lengths and a Y-junction combiner.

To demonstrate the effectiveness of the network in equal power distribution, each laser path was illuminated separately and the photocurrent after each photodiode was measured. This measurement was carried out for two different chips. The maximum photocurrent observed across both chips was then used to normalize the results in order to show the optical power mismatch between the photodiodes and demonstrate repeatability and tolerance to fabrication variation from chip to chip. Supplementary Fig. 2 summarizes the findings of these measurements and show that the variation/mismatch generally remains under 1dB. The only exception is when light is illuminated from the blue (row) with PD1 and PD16 exhibiting less than 1.5dB variation. However, this slightly higher variation is consistent across the two chips and thus suggests that the directional coupler at the end of the bus waveguide needed a slightly longer coupling length.

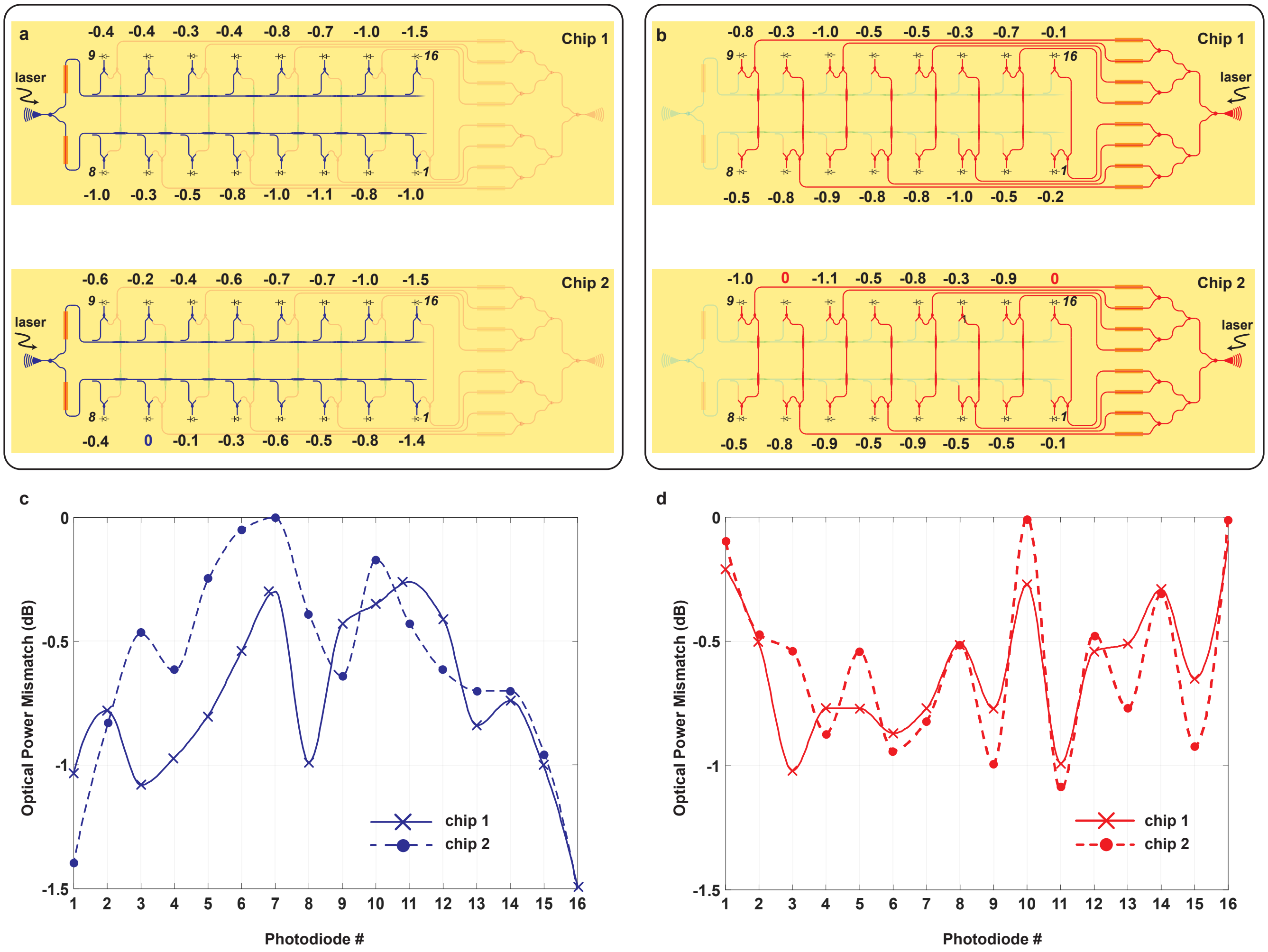

Supplementary Fig. 2 | Optical power distribution mismatch. Normalized detected optical power variation between photodiodes of two chips, shown by location, when light is coupled in through the **a** row path, distributed using cascaded directional couplers with varying lengths, and **b** column path distributed using 4 layers of Y-junctions. The plot of detected optical power variation between the photodiodes when light is coupled in through the **c** row path and **d** column path.

Supplementary Note 3: On-PCB antenna array design

The 2×8 patch antenna array and the distribution lines were designed and simulated using Ansys HFSS 3D electromagnetic simulator. Supplementary Fig. 3a shows the structure of the patch antenna implemented on the 4-layer Rogers 4350 printed circuit board. The design is similar to a previously reported antenna[2] but scaled to 28GHz. Supplementary Fig. 3b shows the cross-section (stackup) of the PCB. The radiation pattern of a single patch antenna is shown in Supplementary Fig. 3c whereas Supplementary Fig. 3d shows the radiation pattern of the 2×8 array including the effect of the distribution lines from the chip to the antennas (for $\theta = 0°$, $\varphi = 0°$ steering angle). The array achieves a simulated gain of around 15.1dB which takes into account the effect of the feed lines, mismatches, and losses. Microstrip transmission lines were used for the mm-wave distribution on layer L1 whereas the patch antennas were printed on L4 with L2 and L3 used as ground planes.

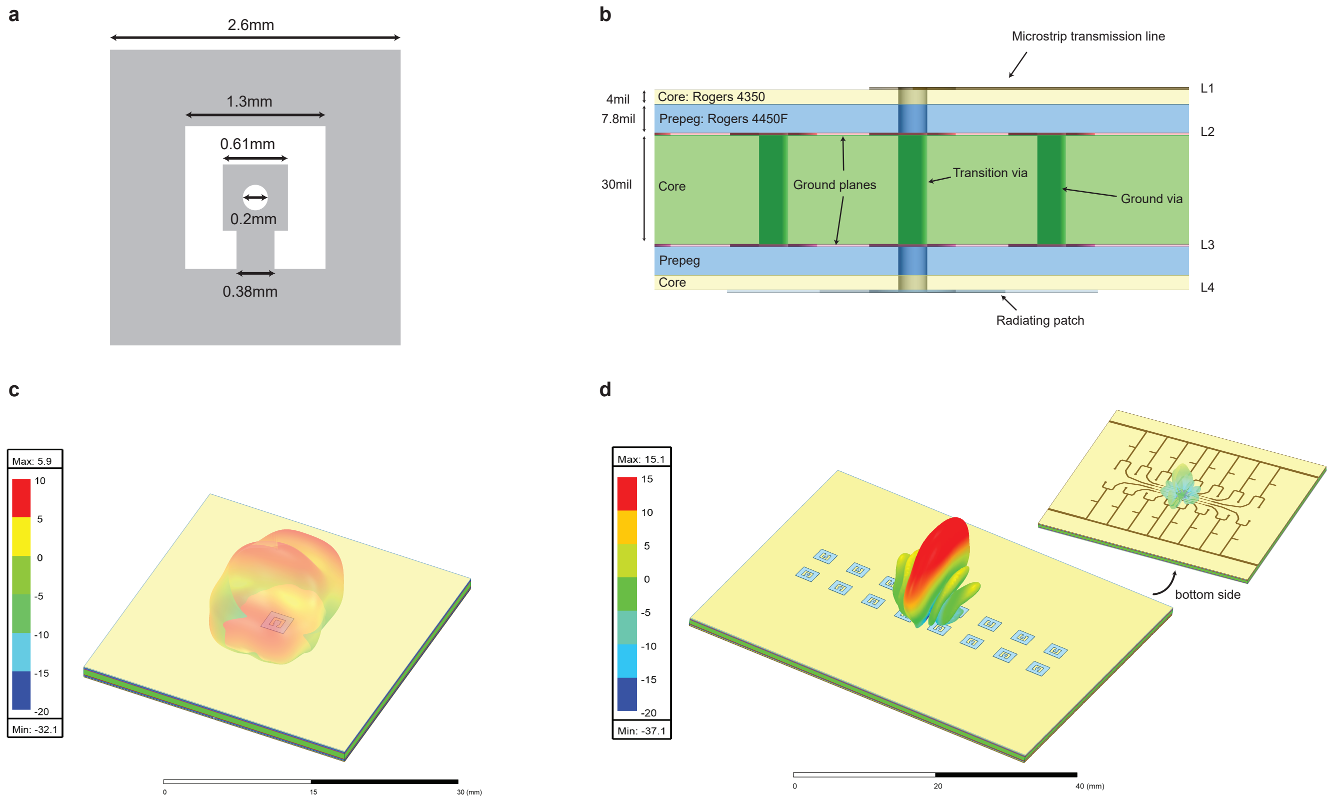

Supplementary Fig. 3 | Printed circuit board simulation and design. a Dimensions of a single patch antenna. **b** 4-layer stackup of the Rogers 4350 PCB with patch antennas and microstrip distribution lines. **c** Radiation pattern of a single patch antenna. **d** Radiation pattern of the 2×8 array ($\theta = 0°$, $\varphi = 0°$).

Supplementary Note 4: Characterization of resistive heaters

Resistive heaters based on doped silicon are used to control the phase of the optical signal in the silicon nanowaveguides using the thermo-optic effect. Such heaters are often characterized by the power required (P_π) to induce a π phase shift in the optical signal. In order to characterize the heaters and determine the P_π, two heaters were placed in the arms of a Mach Zehnder Interferometer (MZI) as shown in Supplementary Fig. 4a. The output of the MZI was photodetected and the photocurrent (I_{PD}) was monitored. The voltage applied across the heater (V_{HEATER}) was then swept and the current flowing through the heater (I_{HEATER}) was measured. Supplementary Fig. 4b also shows a plot of I_{PD} vs. dissipated heater power ($V_{HEATER} \times I_{HEATER}$). A power of roughly 60mW is required to induce an optical π phase shift. It is worth mentioning that the power consumption and hence the efficiency of the thermal phase shifters can be considerably improved by using undercut cavities[3].

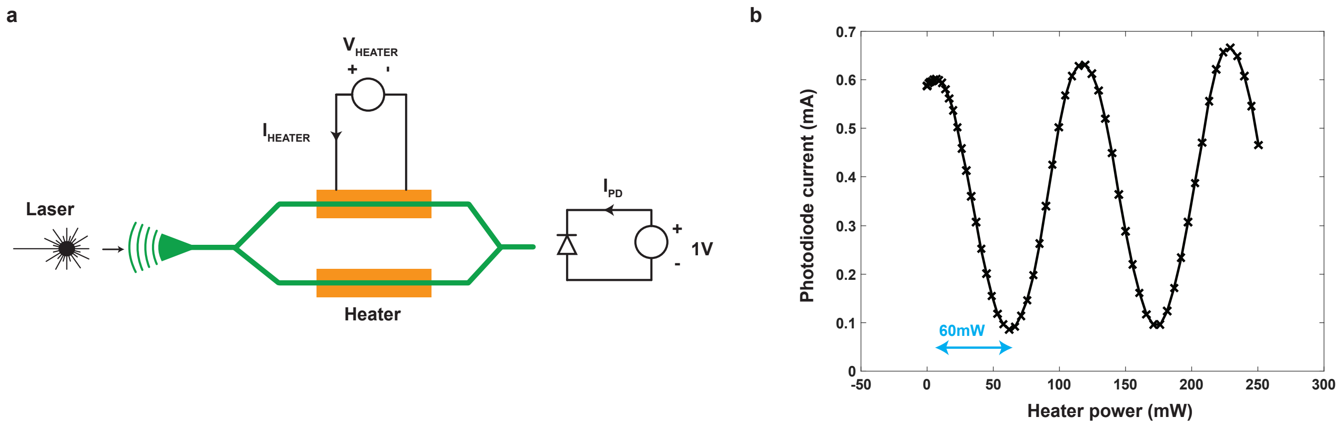

Supplementary Fig. 4 | P_π measurement setup and results. a Experimental setup used to measure the P_π of the doped silicon heaters. **b** Plot of the photocurrent at the output of an MZI vs. dissipated heater power.

Supplementary Note 5: Lateral SiGe photodiode characterization at different reverse biases

Supplementary Fig. 5 shows the characterization results of the SiGe PIN photodiode at different reverse bias voltages. Note that some of the performance metrics have been previously reported in Supplementary ref.[3] (specifically, EO bandwidth of around 42GHz, responsivity close to 1AW^{-1} and dark current of 35-70nA at 1V reverse bias) which are in agreement with the results shown below.

Supplementary Fig. 5a shows the measured response of the photodiode where the bandwidth is lowered from around 44GHz to 40GHz when the reverse bias changes from 1.5V to 0.5V.

Supplementary Fig. 5b is the optical saturation power measurement. In this case, light is coupled into a waveguide through a grating coupler and the DC photocurrent is measured as the optical power is increased. At a reverse bias voltage of 1.5V, saturation occurs at an optical power of around 6mW. Note that the x-axis corresponds to the amount of power coupled into the waveguide and the 5dB loss of the grating coupler has been de-embedded. From this measurement, a responsivity of approximately 1AW^{-1} is also estimated.

Supplementary Fig. 5c shows the measured photodiode output RF power as function of photocurrent which can be used to calculate the compression point of the photodiode as shown in Supplementary Fig. 5d. The results indicate that for a reverse bias voltage of 1.5V, the 1dB compression point occurs at a photocurrent of 1.75mA which reduces to around 1.4mA when the reverse bias voltage is lowered to 0.5V. As a reference point, in the implemented photonic-assisted mm-wave transmitter, with 55mW of coupled optical power (resulting in an EIRP of 5dBm), each of the 16 photodiodes has a photocurrent of around 1.2mA.

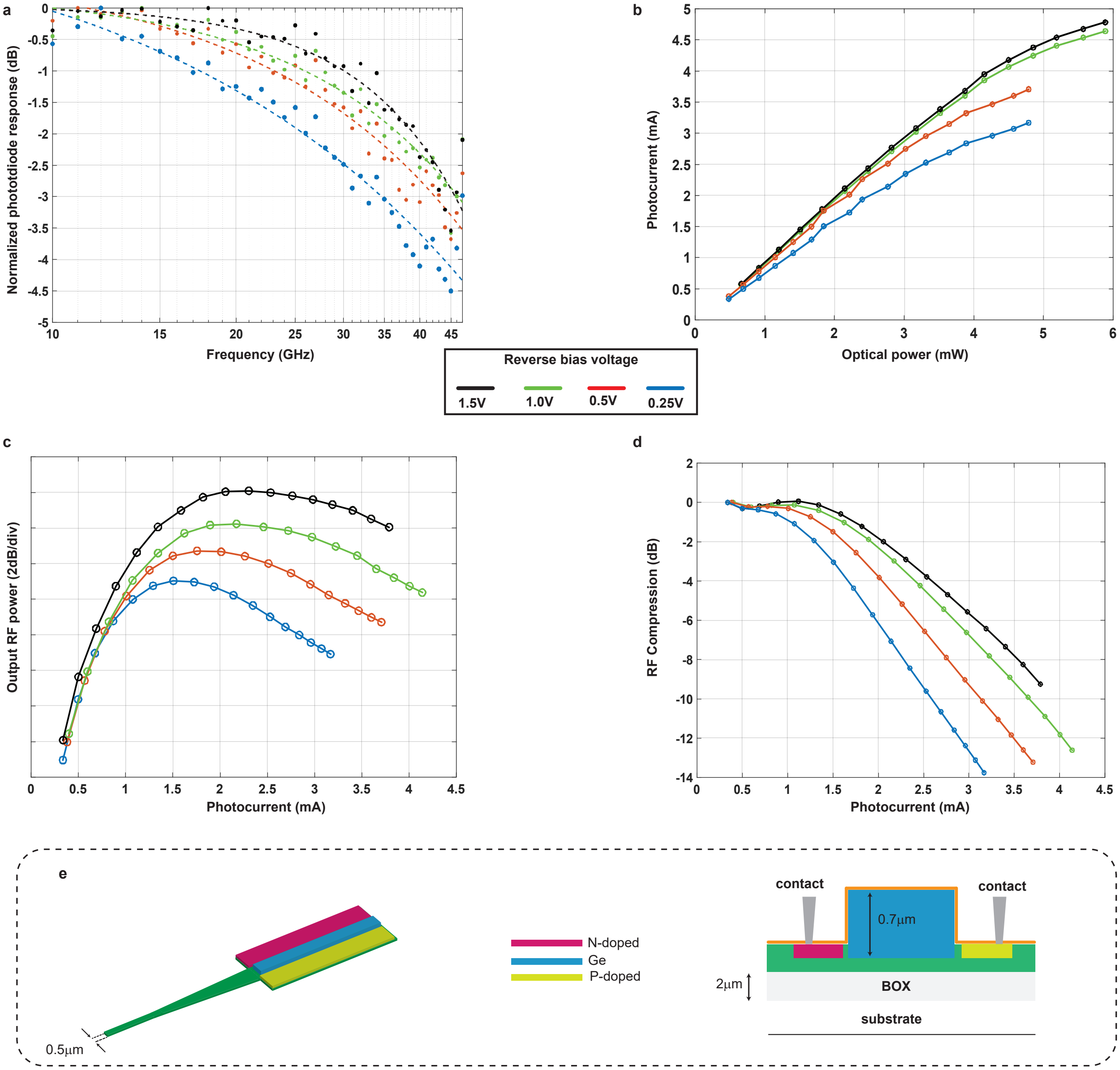

Supplementary Fig. 5 | Photodiode characterization at different reverse biases. a Measured bandwidth of the SiGe photodiode. **b** Measured DC photocurrent vs input optical power. **c** Measured output RF power, **d** compression vs photocurrent at 25.5GHz. **e** Photodiode structure and cross-section.

Supplementary Note 6: Architecture and schematic of the on-chip electronics

Supplementary Fig. 6 shows the schematic of the on-chip electronics used to control the photonic-assisted phased array transmitter chip. 5-bit current-steering digital to analog converters (DAC) were used to set the current flowing through the resistive heaters serving as the thermo-optic phase shifters. The DAC registers were implemented using cascaded flip flops (FF) forming shift registers and programmed externally using a CLOCK signal and DATA bit stream.

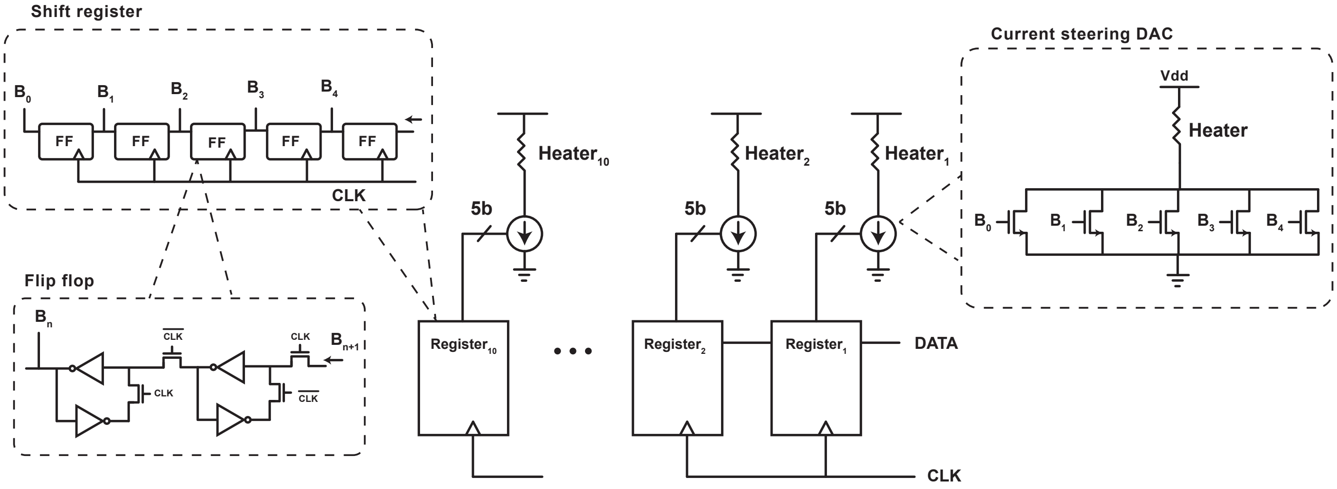

Supplementary Fig. 6 | Schematics of the integrated electronics. Circuit diagram of the electronics used to control the resistive heaters used as thermo-optic phase shifters. Current-steering digital to analog converters (DAC) with binary weighted transistors controlling the current flowing through the heaters. The gates of the DAC transistors are set using 5-bit shift registers. Each shift register consists of five cascaded flip flops.

Supplementary Note 7: Beam formation optimization algorithm

Supplementary Fig. 7a shows the pseudocode of the simple algorithm used for beam formation optimization. The receiving horn antenna is placed at the desired spherical location where the beam should be directed towards. The 10 on-chip 5-bit DACs controlling the heaters are sequentially swept (by increasing the current through the corresponding heaters) and the received power is simultaneously measured. The DAC code that corresponds to the maximum received power is then saved onto that DAC before proceeding to sweep the next DAC. Supplementary Fig. 7b shows a sample optimization demonstrating how the received power changes after each step. Specifically, in this case, the horn antenna was placed perpendicular to the array at elevation and azimuth of 0°. At the end of the optimization process, the DAC settings corresponding to the target angle are saved on a look up table. Successful beam formation is confirmed by measuring the radiation pattern.

a

```
Place horn antenna at desired elevation & azimuth
Initialize DACs
while i < interation count do
    for DACn do
        for data := 0 to 2^5-1 do
            DACn <—— data
            Measure & store received power
        end for
        Pick data_MAX corresponding to maximum received power
        DACn <—— data_MAX
    end for
end while
```

b

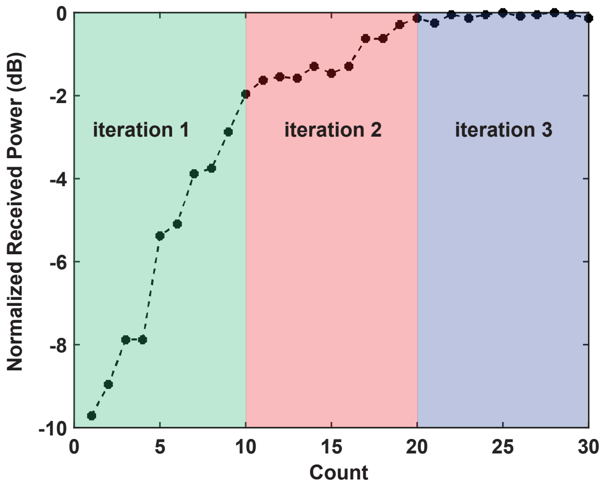

Supplementary Fig. 7 | Beam optimization. **a** Pseudocode of the simple algorithm used for beam formation optimization. **b** The measured received power (normalized) at each step of the optimization.

Supplementary Note 8: 3D radiation pattern measurement procedure

In the radiation pattern measurements, the radiating elements (PCB patch antennas) are stationary. The receiving horn antenna is attached to a robotic arm and placed 31cm away (within the far-field) from the center of the 2$\times$8 antenna array. The robotic arm then moves such that the horn antenna scans the surface of a half-sphere in elevation (θ) and azimuth (φ). In order to measure the 3D radiation pattern of the phased array antenna and generate the plots of Fig. 5c of the main text, the horn antenna's H-plane was first aligned in the x-direction (Supplementary Fig. 8a). The received power was then recorded at different elevation (from $\theta = -40^\circ$ to $\theta = 40^\circ$) and azimuth (from $\varphi = -90^\circ$ to $\varphi = 90^\circ$) angles (Supplementary Fig. 8c and Fig. 8d). The horn antenna was then rotated by 90° so that the H-plane was aligned in the y-direction (Supplementary Fig. 8b) and the measurement process was repeated. For the two antenna configurations, the measured powers at each location (θ, φ) were added and plotted over the specified space.

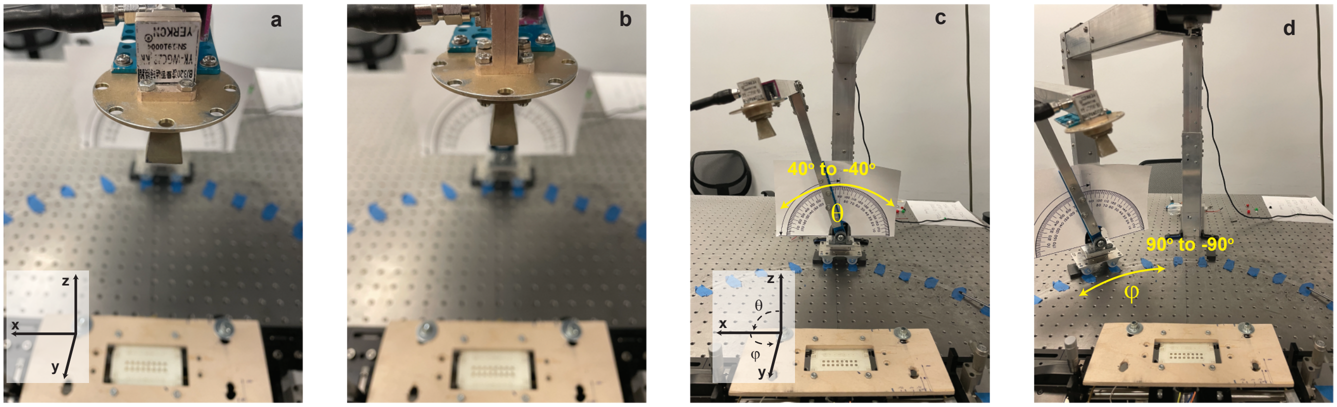

Supplementary Fig. 8 | Antenna alignment and scanning for 3D radiation pattern measurements. Antenna alignment and scanning for 3D radiation pattern measurements. **a** Receiver horn antenna with its H-plane aligned in x-direction. **b** Receiver horn antenna with its H-plane aligned in y-direction. **c** Horn antenna scanning in elevation (θ). **d** Horn antenna scanning in elevation (θ) and azimuth (φ).

Supplementary Table 1

Equipment	Model
Spectrum analyzer	HP 8565E, Agilent PXA N9030A
Wide-bandwidth oscilloscope	Agilent Infiniium DCA 8610A
Bit pattern generator	SHF BPG 40 A
Network analyzer	Agilent E8361A
Signal generator	Anritsu MG3697C
Error analyzer	SHF EA 40 GIG
Power meter	Agilent E4418B
Power sensor	HP R8486D
Power supply	Agilent E3631A, HP E3620A
Power sensor	HP R8486D
Digital multimeter	Agilent 3478A
Crystal detector	Hughes 44820H-500
Amplifier	Mini Circuits ZX60-6013E0S+, Miteq 166787-3, Picosecond x5882
Bias tee	Picosecond Pulse Labs, Anritsu 48383
Laser	HP 81682B, Koheras 1FL01W1EB-AD
Laser current source	LDX-3620
Laser temperature controller	Thorlabs TED 200C
Erbium doped fiber amplifier	Optilab I-24-B, Oprel/JDS Uniphase OFA20L
Optical intensity modulator	UTP 10Gb/s, Thorlabs LN05S-FC

Supplementary Table 1 | List of instruments and components used in the measurement and characterization setups.

Supplementary References

1. Ashtiani, F. & Aflatouni, F. N×N optical phased array with 2N phase shifters. *Opt. express* **27**, 27183–27190 (2019).
2. Waterhouse, R. & Novak, D. Center-fed patch antenna with low cross polarization levels. In *2014 IEEE Antennas and Propagation Society International Symposium (APSURSI)*, 1853–1854 (IEEE, 2014).
3. Giewont, K. *et al.* 300-mm monolithic silicon photonics foundry technology. *IEEE J. Sel. Top. Quantum Electron.* **25**, 1–11 (2019).
